# Vegetation Analysis and Environmental Relationships of Riverain Plants in the Aswan Reservoir, Egypt

**DOI:** 10.3390/plants10122712

**Published:** 2021-12-10

**Authors:** Ahmed M. Abbas, Fatma A. A. Ayed, Mohamed G. Sheded, Sulaiman A. Alrumman, Tarek A. A. Radwan, Mohamed O. Badry

**Affiliations:** 1 Biology Department, College of Science, King Khalid University, Abha 61413, Saudi Arabia; ahhassan@kku.edu.sa (A.M.A.); salrumman@kku.edu.sa (S.A.A.); 2 Department of Botany & Microbiology, Faculty of Science, South Valley University, Qena 83523, Egypt; 3 Department of Botany, Faculty of Science, Aswan University, Aswan 81528, Egypt; fatma_adel@sci.aswu.edu.eg (F.A.A.A.); msgaber1960@yahoo.com (M.G.S.); radwantarek@aswu.edu.eg (T.A.A.R.)

**Keywords:** CANOCO, two dams, life-form, Nile River, riparian vegetation, soil analysis, TWINSPAN, water fluctuation

## Abstract

The present study analyses plant diversity and evaluates the relationship between edaphic variables and the distribution and grouping of plant species in the Aswan Reservoir area, South Egypt. The dominant families were Fabaceae, Poaceae, and Asteraceae, forming 38.82% of the total flora recorded. The main bulk of the flora recorded (50.59%) belonged to the cosmopolitan, neotropical, pantropical, and palaeotropical chorotypes. A TWINSPAN analysis produced 10 vegetation clusters. Inundation levels showed a high correlation with species richness. The seasonally inundated area in Bute El-Hasaya and Maezana Belal (cluster V) had the highest species richness (36.50), while the lowest species richness (4.50) was in the shoreline of Philae, Awad, and Heisa islands (cluster IX). The DCA ordination depicted the environmental gradient expressed by the cluster analysis, and the resulting vegetation groups represented a distinct microhabitat. The CCA ordination indicates that the separation of vegetation group (A) along the axis was affected by the concentration of K, Mg, and CO_3_, and the vegetation group (B) was significantly associated with the total dissolved salts and the concentration of Cl. Moreover, the vegetation group (C) correlated significantly with pH, electrical conductivity, organic matter content, and SO_3_, HCO_3_, PO_4_, Na, and Ca concentrations.

## 1. Introduction

Damming changes hydrological regimes quickly and alters aquatic environments leading to habitat fragmentation, sediment transport, and species migration in regulated rivers [1,2]. It also increases the residence time of water in reservoirs and the surface area receiving solar radiation and decreases the wetland area below the dam [3]. Thus, damming has pronounced effects on wetlands, particularly in arid regions, where river regulation and diversions’ hydrological and ecological effects are acute [4]. Dams are often constructed despite not enough environmental impact assessments. These assessments typically only include an evaluation of water quality and often focus on hydrological connectivity but rarely extend beyond the surrounding dam construction ecosystems [5].

Wetlands are ecologically important due to their hydrologic attributes and role as ecotones between terrestrial and aquatic ecosystems. They function as downstream recipients of water and waste from natural and human sources and have often been transformed into drylands for agriculture and human settlements [6]. Moreover, river control schemes have usually caused the loss of wetlands [7]. Changes in the environmental conditions would result in an ongoing evolution of the native plant cover. These changes may explain the high complexity of the weed flora of Egypt, where some plants, which were once common, are now rare or almost extinct. Moreover, several exotic species introduced in different ways are now stabilized and naturalized [8].

Nevertheless, river regulation and dam construction are strongly altering natural riparian habitats and pose high ecological risks. These changes may have direct or indirect effects on plant diversity in riparian wetlands [9]. For most plant habitat studies, the relationship between soils and vegetation is significant. Vegetation is an indicator of the soil’s physical and chemical properties and can maintain a balance of nutrient levels in the water body [10]. Studying these properties could be an effective and useful tool to promote and facilitate the transfer of information about these lands from scientists to land managers [11].

The major environmental factors correlated with vegetation patterns are often presented as descriptive documentation of species and their classification [12]. A more subjective methodology for local and often regional scale use has been favored by modern synecological research, trying to summarise broad complex data sets either by classifying or ordering floristic data and then linking the results to environmental data, or deriving plant habitat relationships from a single analysis of a combined floristic and environmental variable set. 

The Nile River and its tributaries have been subjected to huge environmental changes caused by the construction of dams and barrages. Examples include the destruction of many natural habitats and artificial ones, such as cultivated fields on river islands, aquaculture plots, and the extensive spread of aquatic weeds in Egyptian water bodies, particularly in the network of canals and drains in the Nile valley region [13]. Furthermore, these dams and barrages have segmented the natural hydrological system, undoubtedly impacting the biota [14]. 

In Egypt, the Aswan High Dam construction during the 1960s altered the river morphology, including island formation and khors [15]. The formations constituting the zonation from the river edge to the second terrace are controlled by topographic features that regulate the water surpluses and shortages [16]. Before the Aswan High Dam construction, all the Nile Valley and Nile Delta lands were watered by the inundation of the basin system. Thus, the water’s silt gave an annual increase in sediment in all lands of the Nile Valley and Nile Delta, causing an increase in the thickness of substrate by one meter every 1000 years [17]. These silts were carried by the Nile water from Ethiopia through the Blue Nile, forming the good fertile land of black or reddish color in layers with sand. However, upstream dams have changed sediment dynamics and now prevent silt from reaching Egyptian lands [18]. 

Many studies have documented or predicted significant biodiversity losses downstream of dams [19,20,21,22]. Native aquatic species such as fish have decreased in warm-water rivers due to cold, clear water discharges from dams; movement limits are caused by dams, changes in hydrological regime, and disconnections between river and floodplain, among other factors [23]. Most large dams have affected the vegetation considerably in the central and western United States, where populus woods dominate and three distinct patterns emerge [24]. Populus and its closely related species have failed to recover downstream of dams on meandering rivers in cooler climates, leading to a more homogeneous and poorer forest cover [25]. Contrastingly, braided sand-bed rivers, dams, and water diversions have caused channel narrowing and native populus expansion, possibly increasing biodiversity, at least for the short-term [26]. Invasive woody plants have dominated over certain rivers in hot semiarid areas (*Tamarix*, *Elaeagnus*) that are better adapted to river regulation than the native populus, or have colonised rivers with unfavourable natural flow regimes [27]. The rate of biodiversity loss below dams is unknown. Moreover, rapid loss is predicted unless important riverine phenomena such as flow variability and cut-and-fill alluviation are restored [23]. 

Based on the recent floristic study of the Aswan reservoir area [28], the key questions addressed in this paper to enrich ecological knowledge in key riparian zones along the Aswan Reservoir were: (1) what is the current floristic composition of the Aswan Reservoir area?; (2) what are the ecological effects of water-level fluctuations on the Aswan Reservoir area’s vegetation structure?; and (3) what are the edaphic factors that control the distribution of plant communities and species diversity?

## 2. Results

### 2.1. Floristic Composition

A total of 170 species were recorded in the study area, belonging to 139 genera in 46 families (Table S1). Dicots were represented by 39 families and 122 taxa, while six families and 47 taxa represented monocots. The ferns were represented by one family and included only one species (*Adiantum capillus-veneris* L.). Fabaceae (27 species), Poaceae (24 species), and Asteraceae (15 species) were the most species-rich families (Figure S1, Table S1). On the other hand, 21 families were poorly represented, having one species each. The largest families in terms of the number of genera were Fabaceae (21 genera), Poaceae (21 genera), and Asteraceae (14 genera). 

**Figure 1:**
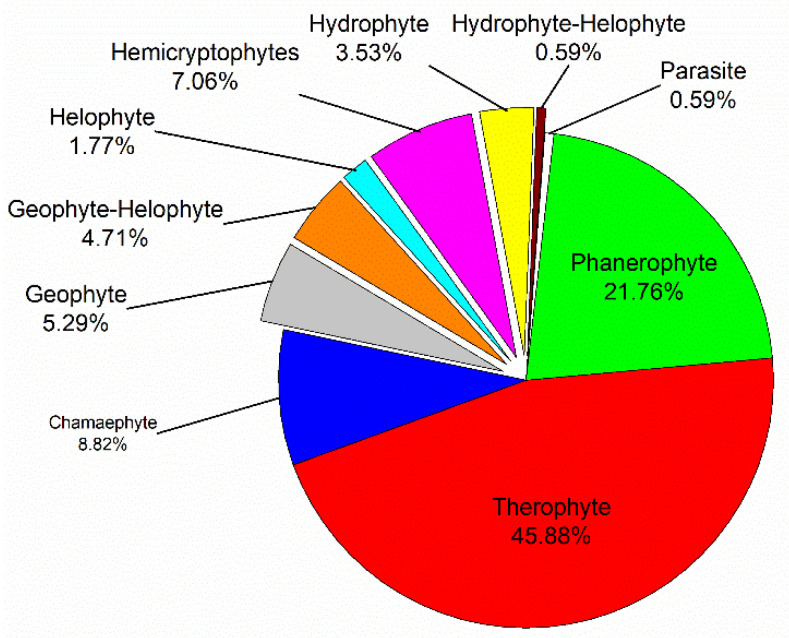
Life form spectrum of the plant species recorded in the Aswan Reservoir area.

Ten life-forms were recorded in the present study. Therophytes were the most abundant life-form (77 species), followed by Phanerophytes (37 species) and Chamaephytes (15 species), while a single species (*Cuscuta pedicellata* Ledeb) was Parasite (Figure 1).

**Figure 2:**
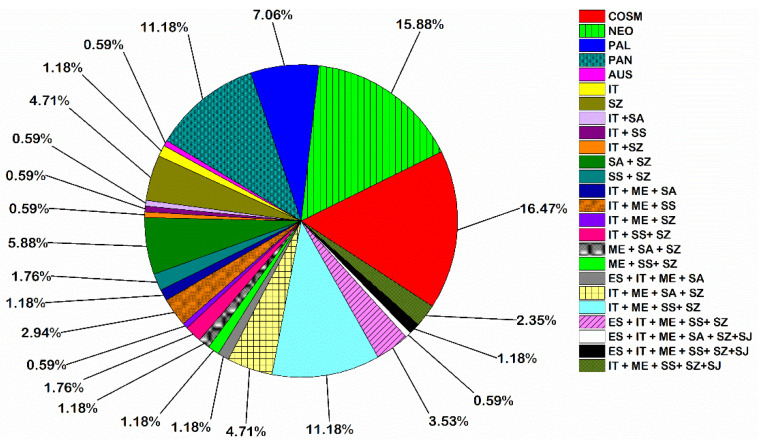
Phytogeographical analysis of plant species recorded in the Aswan Reservoir area. Chorotype abbreviations: AUS: Australian, COSM: Cosmopolitan, ME: Mediterranean, NEO: Neotropical, PAL: Palaeotropical, PAN: Pantropical, ES: Euro-Siberian, IT: Irano-Turanian, SA: Saharo-Arabian, SS: Saharo-Sindian, SJ: Sino–Japonic, SZ: Sudano-Zambezian.

Chorological analysis of the 170 plant species recorded in this study revealed that Cosmopolitan (28 species), Neotropical (27 species), Pantropical (19 species), and Palaeotropical (12 species) chorotypes constituted the main bulk of the total number of plant species recorded (86 species). The pluriregional elements were represented by a total of 57 species of different affinities. These pluriregional species fall under 13 main chorotypes, of which 19 species represented the Irano-Turanian/Mediterranean/Saharo-Sindian/Sudano-Zambezian chorotypes. Sixteen species represented the bi-regional chorotype. Ten species originally came from the Saharo-Arabian/Sudano-Zambezian chorotype. On the other hand, the mono-regional chorotype was represented by 11 species, of which eight were Sudano-Zambezian (Figure 2).

### 2.2. Distribution of Families among the Surveyed Locations

**Figure 3:**
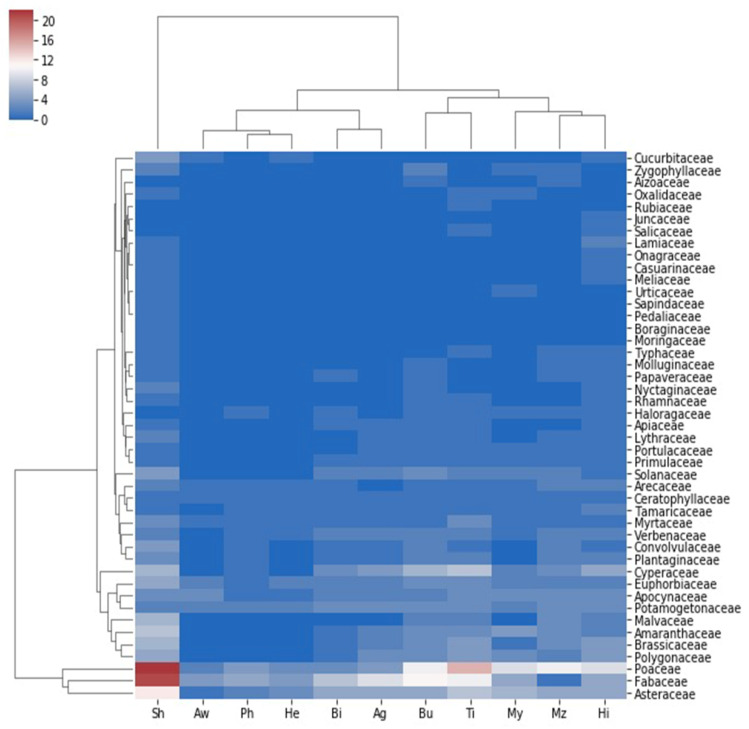
Heatmap illustrating the distribution of families on the different locations surveyed at the Aswan Reservoir area. Sh: El Shallal area, Aw: Awad Island, Ph: Philae Port, He: Heisa Island, Bi: Bigga Island, Ag: Agilkia Island, Bu: Bute El-Hasaya, Ti: Tingar, My: El Mahgar Valley, Mz: Maezana Belal, Hi: High Dam Colony.

The surveyed plant families exhibited a distinct distribution pattern concerning the different habitats within the Aswan Reservoir area, forming two groups. One (group I) included species-rich families Fabaceae and Poaceae. In contrast, the other (group II) had all other families. However, the El Shallal separated as one group regarding the surveyed locations, while the other sites surveyed were in the other group (Figure 3).

### 2.3. Similarity Coefficient between the Investigated Sites in the Aswan Reservoir Area

A high similarity (positive correlation) was observed between the sites of Heisa and Awad (*r* = 0.90), Agilkia and Bigga (*r* = 0.91), the High Dam and Bute (*r* = 0.81), El Shallal and Tingar (*r* = 0.61), and Philae and Awad (*r* = 0.67). However, a low similarity (negative correlation) was detected between the sites of Bigga and El Shallal (*r* = −0.22), Agilkia and the High Dam (*r* = −0.74), and Tingar and Bigga (*r* = −0.62) (Table 1).

### 2.4. Vegetation Analysis

**Figure 4:**
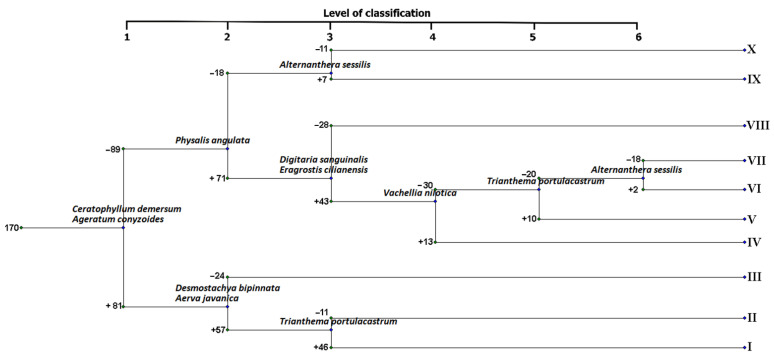
The dendrogram resulting from the application of TWINSPAN on the 27 sampled vegetation stands.

The vegetation of the Aswan Reservoir area was grouped into 10 vegetation clusters at level six of hierarchical classification (Figure 4). TWINSPAN identified one or two indicator species for each cluster. 

The clusters were characterized and named after the dominant and subdominant species as follows: (I) *Phragmites australis*–*Caroxylon imbricatum* (stands of High Dam Colony and El Mahgar Valley); (II) *Imperata cylindrica*–*Pluchea dioscoridis*–*Dichanthium annulatum* (stands of El Shallal area); (III) *Cynodon dactylon*–*Tephrosia purpurea* subsp. *apollinea* (stands of El Shallal area); (IV) *Vachellia seyal*–*Vachellia tortilis* subsp. *raddiana*–*Trifolium resupinatum* (stands of El Shallal area); (V) *Ammannia baccifera*–*Eleocharis parvula*–*Polygonum aviculare* (stands of Bute El-Hasaya and Maezana Belal); (VI) *Lepidium didymum*–*Lepidium coronopus*–*Physalis angulata* (stands of El Shallal and the High Dam Colony); (VII) *Eleocharis geniculata*–*Veronica anagallis-aquatica* (stands of Bigga and Agillkia islands); (VIII) *Ageratum conyzoides*–*Rorippa palustris* (stands of El Mahgar Valley and Tingar island); (IX) *Sesbania sesban*–*Tamarix nilotica* (stands of Philae, Awad, and Heisa islands); and (X) *Ageratum conyzoides*–*Alternanthera sessilis*–*Eleocharis geniculata* (stands of Agillkia island). 

**Figure 5:**
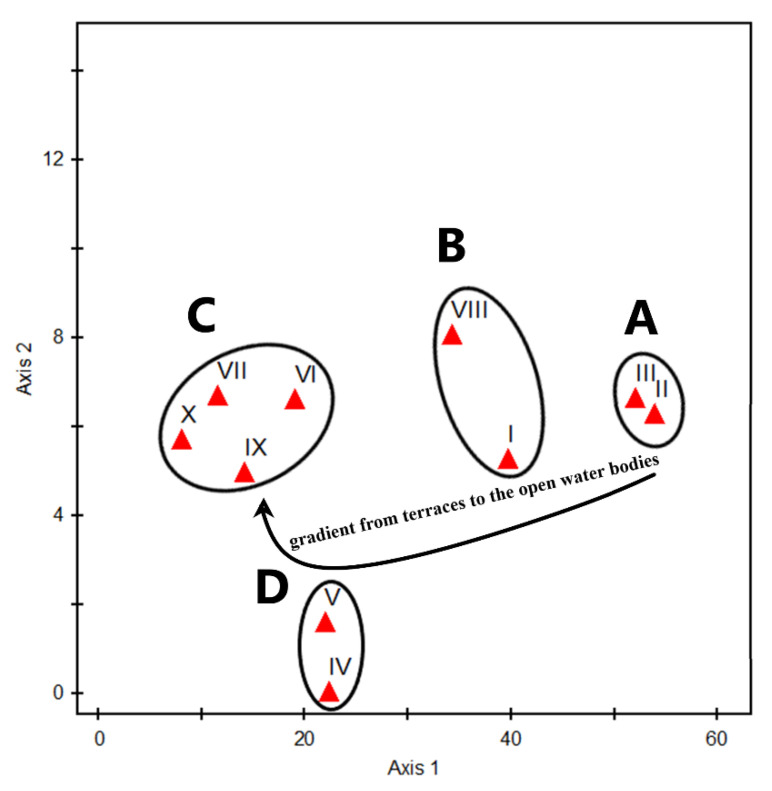
DCA ordination diagram on the first two axes (axis 1 and 2) of the 10 vegetation clusters (red triangles) identified after the application of TWINSPAN on the 27 sampled stands in the Aswan Reservoir area.

The application of a DCA analysis supported the separation among the vegetation clusters. A distinct pattern along the gradient of DCA axes 1 and 2 indicated the relationships between environmental gradients (proximity from water) and topographic aspects of the Aswan Reservoir area. The TWINSPAN clusters were aggregated into four main vegetation groups, which depicts a distinct microhabitat (Figure 5). The floristic group (A) represented cultivated microhabitats and consisted of clusters II and III, ordered along the gradient of DCA-axis 1. Group (B) represented shore and bank slope microhabitats and comprised of clusters I and VIII, ordered along the gradient of DCA-axis 1. Group (C) represented island shore and bank slope microhabitats comprised of four clusters, VI, VII, IX, and X, ordered along the gradient of DCA-axis 2. Group (D) represented shoreline microhabitats and included two clusters, V and IV, ordered along the gradient of DCA-axis 1.

**Figure 6:**
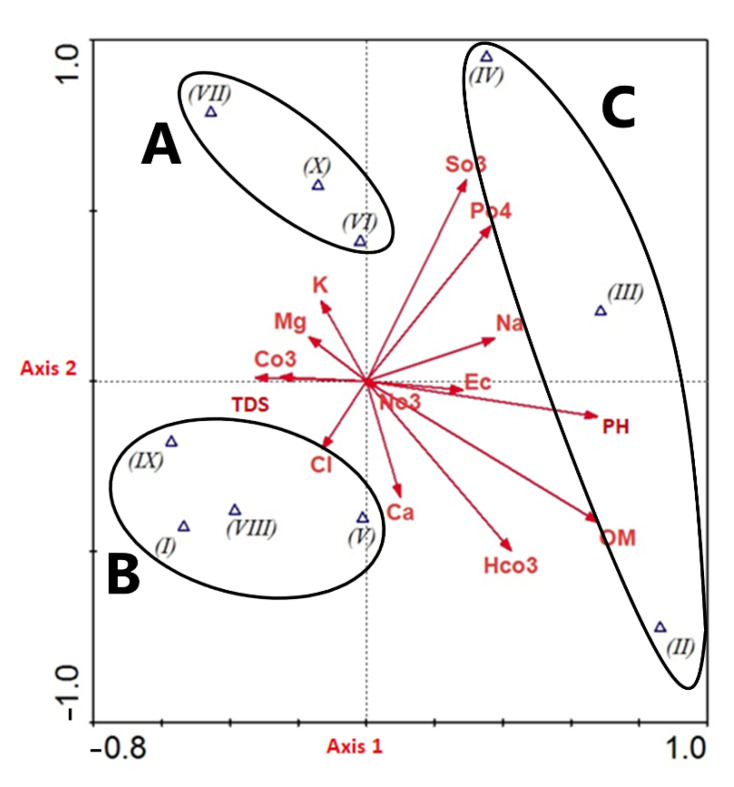
CCA Biplot ordination of the environmental variables (arrows) and the TWINSPAN vegetation groups (blue triangles). TDS: total dissolved salts, EC: electrical conductivity, OM: organic matter, Ca: calcium, Mg: magnesium, K: potassium, Na: sodium, CO_3_: total carbonates, HCO_3_: bicarbonates, Cl: chlorides, SO_4_: sulfates, NO_3_: nitrates, PO_4_: orthophosphates.

In the CCA scatter plot, the ten TWINSPAN clusters were aggregated into three main floristic groups (Figure 6). The separation of these groups along the CCA-axis 1 was strongly affected positively by Na, K, Mg, PO_4_, SO_3_, and CO_3_ and negatively with Ca, HCO_3_, Cl, OM, pH, EC, and salinity. On the other hand, the separation of floristic groups along the CCA-axis 2 correlates positively with Ca, Na, SO_3_, PO_4_, HCO_3_, OM, pH, and EC and negatively with K, Mg, Cl, CO_3_, and salinity. Both axis 1 and 2 were weakly correlated with NO_3_. The CCA group (A) strongly correlated with K, Mg, and CO_3_ concentrations, while group (B) correlated significantly with TDS and Cl. On the other hand, group (C) was significantly correlated with Na, PO_4_, SO_3_, Ca, HCO_3_, OM, pH, EC, and weakly correlated with NO_3_.

### 2.5. Plant Community and Soil Correlation

The *Ammannia baccifera*–*Eleocharis parvula*–*Polygonum aviculare* community (cluster V) demonstrated the highest levels of species richness (36.50), and the *Sesbania sesban*–*Tamarix nilotica* community (cluster IX) had the lowest species richness (4.50). On the other hand, the *Cynodon dactylon*–*Tephrosia purpurea* subsp. *Apollinea* community (cluster III) attained the highest species turnover, while the *Ageratum conyzoides*–*Alternanthera sessilis*–*Eleocharis geniculate* community (cluster X) had the lowest species turnover (1.00) (Table S2).

Five species (*Cynodon dactylon*, *Euphorbia hirta*, *Imperata cylindrica*, *Leptadenia arborea*, and *Tephrosia purpurea*) have broad ecological amplitude and were recorded in ten clusters. Moreover, seven species are present in nine clusters. These species have wide ecological amplitude but seem to prefer certain habitat types; for example, *Cuscuta pedicellata, Phoenix dactylifera*, and *Pluchea dioscoridis* are not recorded in cluster X but are well represented in the rest of the clusters. *Phragmites australis*, *Sesbania sesban*, and *Psidium guajava* are not recorded in clusters II but are well represented in the rest of the clusters. Most of the species showed preference to certain habitat types; for example, *Portulaca oleracea* and *Trigonella hamosa* have ecological success in the cultivated areas of these islands, while *Calotropis procera*, *Tamarix senegalensis,* and *Dichanthium annulatum* high presence value in the cluster indicates ecological success in xerophytic habitat conditions. Twelve species are present in six clusters, and nineteen species are present in five clusters. Most of these species showed preference to certain habitat types; for example, *Persicaria decipiens*, *Cyperus longus*, *Eleocharis geniculata*, and *Rorippa palustris* are confined to moist banks of islands. However, *Chenopodium album, Chenopodium murale, Malva parvifloram, Digitaria sanguinalis*, and *Echinochloa colona* have high presence values in habitats with mesophytic conditions. Twenty species are present in four clusters, and twenty-six species are present in three clusters. Certain species of this group showed a preference habitat conditions; for example: *Amaranthus spinosus*, *Ammannia baccifera*, *Lepidium coronopus Eleocharis parvula,* and *Rumex dentatus* were confined to the wet-bank shore littoral habitats. However, the areas inhabited by farmers contain some of the shade trees and shrubs, e.g., *Syzygium cumini, Acacia nilotica, Casuarina equisetifolia, Acacia seyal, Bougainvillea glabra*, and *Khaya senegalensis.*

Moreover, there are some cultivated plants, such as *Corchorus olitorius*, *Eruca sativa* and *Lactuca sativa*, in addition to weeds associated with crops, for example, *Argemone mexicana*, *Cyclospermum leptophyllum*, *Dactyloctenium aegyptium*, *Lotus arabicus*, *Oxalis corniculata*, and *Trianthema portulacastrum*. Twenty-five species are present in two clusters and thirty-seven species are present in one cluster; these species have a limited ecological range and indicate a clear preference for certain habitats. 

Habitats of the studied stands are composed of different soil fractions. Sand represents the highest part among other soil fractions. It ranged from 10% at a 25 cm depth in cluster I and reached 100% in different clusters. The silt was high in clusters II and III, comprising 20% and 30% of the soil texture, respectively, while gravel was represented by small amounts ranging between 5% and 20%. Clay had the lowest ratio among other soil particles. Only two clusters, VII and X, were devoid of clay. Moreover, the organic matter content in these stands was relatively constant in most clusters. It ranged between 0.573% and 3.57% (Figure S2). 

Variation in the edaphic factors was detected among stands of the Floristic groups (Figure S3, Table 2). The highest value of the total dissolved salts was attained in cluster X (0.05 mg/L), while cluster IX attained the lowest value (0.017 mg/L). Cluster X gained the highest electrical conductivity (EC) mean value (205 μs/cm), while the lowest mean value was detected in cluster V (103 μs/cm). Na concentration attained the highest value in cluster V (29.9 mg.g^−1^) and the lowest in cluster VIII (9.71 mg.g^−1^), while Ca content attained the highest concentration in cluster II (0.833 mg.g^−1^) and the lowest in cluster X (0.467 mg.g^−1^). The Mg content reached the highest concentration in cluster VII (0.52 mg.g^−1^) and the lowest concentration in clusters II and V (0.34 mg.g^−1^) (Table 2).

## 3. Discussion

In terms of the floristic diversity of the study area, 170 species were recorded belonging to 139 genera and 46 families of vascular plants, along with one family of ferns (Pteridaceae) represented by only one species (*Adiantum capillus-veneris* L.). The floristic analysis showed that five major specie families comprised 53.53% of the total flora surveyed in the study area, while three of these (Asteraceae, Fabaceae, and Poaceae) were reported as dominant in the floristic structure of the River Nile and other parts of Egypt [29,30,31,32,33,34]. Compared to the floristic diversity of other Riverain regions in Aswan, the number of species recorded in this study (170 taxa) is within the range, since 94 species of vascular plants were recorded by [35] in the first Cataract, 206 species were recorded in seven islands in the Nile stream north of the Aswan dam until reaching Edfu [36], and 162 species were recorded in ten islands in the River Nile area between Aswan and Esna [37]. However, the floristic composition of these areas varies concerning the dominant plant families.

Asteraceae, Fabaceae, and Poaceae have the widest distribution in the El Shallal area, among other families. This distribution results from the combination of efficient long-distance dispersal, successful establishment, ecological flexibility, disturbance tolerance, and the ability to change ecosystems by modifying the dynamics of fire and mammalian herbivory [38]. Furthermore, these three families were also reported as the most frequent in eastern Ethiopia, northern Zambia, the Mediterranean, and North Africa [39]. This distribution might result from their varied ecological range of tolerance and seed dispersal efficiency over local conditions of water depth [40]. 

The flora of the study area has a high level of monotypism. Among the 46 families recorded, 22 (47.83%) were represented by only one species. Moreover, 119 genera (85.61%) were monotypic, which might be a consequence of the existence of the Egyptian flora in the category of a widespread mid-continental flora having a low level of speciation and a high level of monotypism [41]. Moreover, our results match the findings of [36], who reported 145 (69.2%) monotypic genera for the entire flora of the Egyptian Nubia. This high level of monotypism may be because few plants tolerate the harsh environments in these areas, such as the severe physical disturbance in the Aswan Reservoir caused by the daily water fluctuations. The floristic diversity of the Aswan Reservoir area is 1.22 (170/139) taxa per genus, a ratio less than 2.78 (2100/755), which was recorded for the total flora of Egypt [42]. This remarkable diversity may be due to substrate discontinuities, water availability, water fluctuations, mosaic habitat, topographic diversity, and prolonged human interference.

The life-form spectrum of the Aswan Reservoir flora is dominated by therophytes (45.88%), followed by phanerophytes (21.76%). This trend is similar to the whole Egyptian flora and corresponds to the vegetation spectra of other riverain habitats in Egypt [33,34,43,44,45,46]. The dominance of therophytes may be attributed to various factors such as their short life cycle and high growth rate which enables them to resist substrate instability, in addition to their genetic and morphological plasticity under the high level of disturbances, such as water fluctuations, hot and dry climate, topographic variation, biotic influence, and human activities [7].

Chorological analysis of the 170 species recorded in this study revealed the Cosmopolitan, Neotropical, Pantropical, and Palaeotropical chorotypes (16.47%, 15.88%, 11.18%, and 7.06%, respectively) constituted the main bulk of flora of the Aswan Reservoir area (50.59%). These results agree with the findings of [47]. Elements of the Sudano-Zambezian chorotype are the most dominant chorotypes (70 species = 41.18% of the total flora recorded), including eight monoregionals, 14 biregionals, and 48 pluriregionals. In comparison, the Mediterranean chorotype was represented by 54 species (31.76%) of pluriregional origin. These results were in line with other studies of the flora of upper Egypt and Nubia, where the Sudano-Zambezian elements exceed that of the Mediterranean ones in the entire flora [33,36]. The low number of Mediterranean species may be attributable to the narrow alluvial strips coupled with a dry and hot atmosphere in the study area, allowing only a very limited movement of Mediterranean species [29]. Moreover, the biregional and pluriregional elements were highly represented (42.94% of the recorded plants) in the floristic structure in the study area. This mixture of different floristic elements represented by a variable numbers of species can be attributed to various factors such as water fluctuations, history of farming, and the ability of certain species to penetrate the study area from several adjacent phytogeographical regions [33].

The number of recorded species and their presence in the Aswan Reservoir area differ from one location to another, with neighboring locations showing remarkable differences in their floristic composition. Species richness is highest in the shorelines (El Shallal, Maezana Belal, Tingar, and High Dam Colony) subjected to flooding, which may be due to the strong heterogeneity of these environments compared with those of islands in the study area (EL-Heisa, Philae Port, and Awad islands). Moreover, [48] stated, “wet locations had higher species richness than the dry ones.” On the other hand, the high similarity observed between the locations of Heisa and Awad, Agilkia and Bigga, the High Dam, and Bute El-Hasaya (Table 1) might be due to their close geographical position and exposure to the same conditions since they are currently uninhabited islands [49]. However, the very low similarity reported between Bigga and El Shallal, Agilkia and the High Dam, and Tingar and Bigga might be due to the different habitat, different human impact, distinct topography, and landform patterns, and the large distance between these locations, which has an influence on their abiotic factors and in turn on their floristic similarity [50]. 

The application of TWINSPAN classified the vegetation of the Aswan Reservoir area into 10 vegetation clusters. Cluster V (*Ammannia baccifera*–*Eleocharis parvula*–*Polygonum aviculare* community) had the highest species richness (36.50). This cluster is seasonally inundated in Bute El-Hasaya and Maezana Belal, and farmers exploit these areas in cultivation during the winter when these stands are exposed. Thus, the high species richness of this cluster could be attributed to human practices, such as the transference of weed seeds from other cultivated areas in Egypt [33]. Excessive nutrient loading has recently occurred from an increased discharge of household wastewaters, as well as non-point pollution from agricultural activities and urban expansion, which is one of the key reasons for reservoir eutrophication, which accounts for increasing species richness [51]. Moreover, local variations in topography and substrate heterogeneity of these stands might directly affect their species richness. These findings are in line with studies indicating that many annuals dominate the vegetation of disturbed stands and, therefore, account for increased species richness [50].

On the other hand, cluster IX (*Sesbania sesban*–*Tamarix nilotica* community) had the lowest species richness (4.50). The stands of this cluster are located in Philae, Awad, and Heisa islands and represent shore habitats, so the low species diversity of this cluster may be related to the shallow soil depth associated with the fluctuation of the water level and cleaning practices [52]. Moreover, most of the species in this cluster are specific to aquatic habitats. Thus, species replacement or biotic exchange is low [53]. It is noticed that the dense canopy of tall-growing species along the water edge (e.g., *Phragmites australis*) makes the germination and growth of other species more difficult, often leading to the reduction in the species diversity [54]. 

The first and second DCA ordination axes 1 and 2 depicted the environmental gradient expressed by the cluster analysis (Figure 5). The TWINSPAN clusters were aggregated in four main vegetation groups, representing distinct microhabitats. Among these floristic groups in the study area, vegetation group (A) represents cultivated areas and has a human impact. Group (B) represents the slope bank habitats and sanitation areas opposite the desert, while groups (C) and (D) represent the flood plain microhabitats. These groups show a vegetation gradient from species of cultivated lands at one end (right side) to species of open water zones at the other end (left side). The relationships between these groups may be due to the close similarities of their floristic composition and natural habitats [55]. Furthermore, in some cases, the distribution of some species overlaps other groups due to a wider ecological niche of these species [56]. 

The CCA ordination indicates that species diversity in the vegetation group (A) correlates positively with potassium, magnesium, and carbonates. The clusters of this group (VI, VII, and X) are distributed in El Shallal, High Dam Colony, Bigga, and Agillkia islands, and most of them are shore or seasonally inundated habitats. Soil texture is sand with the lowest silt and clay percentages and low organic matter, sodium, and calcium, which might be due to the seasonal wash of the soil caused by water fluctuation. On the other hand, species diversity in vegetation group (B) correlates positively with the concentration of chlorides and salinity. Clusters of this vegetation group (I, V, VIII, and IX) are present in the High Dam Colony, El Mahgar Valley, Bute El-Hasaya, Maezana Belal, Philae, Awad, Heisa, and Tingar islands. Most of these clusters represent shore habitats with uninhabited impacts. 

However, the vegetation group (C) correlates positively with increasing pH, EC, SO_3_, HCO_3_, PO_4_, Na, Ca, and organic matter. The clusters of this vegetation group (II, III, and IV) are mainly located in the El Shallal area and are cultivated during winter when they are exposed. The crop plants have a major effect on the weed flora in this group. The continuous removal of undesired weed species by farmers in cultivated lands may explain the variability in recorded species among different vegetational groups. 

The high percentage of organic matter may be caused by the decay of the dead plants, which are submerged during the summer flooding after the stands are exposed during winter and have a dense plant cover, which supports the growth of many hydrophytes (*Ceratophyllum demersum* and *Stuckenia pectinata*) and reed swamp plants such as *Phragmites australis*, *Typha domingensis,* and *Paspalum distichum* [33]. Moreover, the soil in these clusters is characterized by high percentages of phosphates, bicarbonates, low potassium rates, and low values of chlorides, which might enhance the growth of weed communities associated with cultivated land [33,36]. Such correlation between the environmental factors and the arrangement of clusters on the two compositional gradients suggests that the distribution of species is governed primarily by these factors [57].

According to a redundancy analysis (RDA) of the plant-soil interactions, the distribution of vegetation in the study area was largely determined by pH, organic matter, calcium, and phosphate contents. The importance of organic carbon in soil fertility is well understood. In the bed of Wadi Araba in the northern part of the Eastern Desert, [58] pointed out the importance of soil organic matter in establishing plant communities. Similar studies have examined the role of different size classes of surface sediments on the geographical distribution of soil moisture [59]. The decay of plant debris elevated the organic matter content in the soil. The dissolved potassium and calcium from rainwaters reduced salt toxicity, resulting in an enrichment of vegetation diversity [60]. Therophytes in the Aswan reservoir area had different reactions to several soil factors, indicating that soil water content, organic matter, silt, pH, Mg, and PO_4_ are the most important environmental requirements. Meanwhile, organic matter, soluble ions, particularly salinity factors (Na and Cl), coarse sand, and fine sand were favoured by the most of perennials (hemicryptophytes, chamaephytes, and phanerophytes). It was obvious from this investigation that annuals dominated the study area, while perennials suited sandy soils.

The investigated sector of the Nile River has been subjected to damming and has been shown to be an ideal ecosystem for a study. The study provided many insights into how a dam and its major hydro-morphological alterations to a river ecosystem can impact vegetation patterns. In addition, analysis of this river sector has enabled us to highlight how the adoption of nature conservation strategies can positively influence vegetation dynamics, ensuring maintenance and protection of the interest of community vegetation types where many vegetation types with high naturalistic importance have developed and expanded after the dam construction. Therefore, damming of the river seems to have had a favorable effect from the conservation viewpoint and biodiversity increase, at least in vegetation.

## 4. Materials and Methods

### 4.1. Study Area

**Figure 7:**
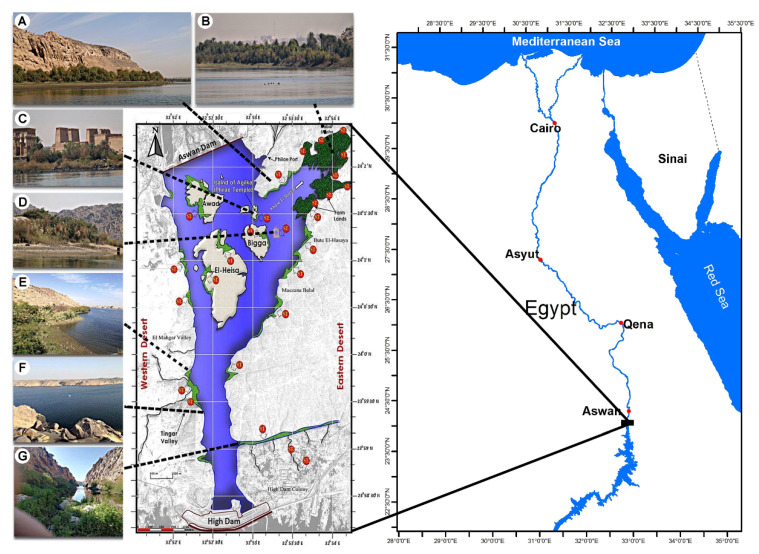
Location map of the study area of the Aswan Reservoir showing the sampling sites. Photos show the landscape of some locations: (**A**) Philae Port, (**B**) El Shallal area, (**C**) Agilkia, (**D**) Bigga Island, (**E**) El Mahgar Valley, (**F**) Tingar, (**G**) High Dam Colony.

The Aswan Reservoir is a lake between two dams: the Aswan High Dam downstream and the Aswan Old Dam at the extreme south of the Nile River in Egypt. The study area extends between 23°58′20″ and 24°02′19″ N and 32°51′50″ and 32°54′08″ E, with a 1.05 km average width and 7.2 km length (Figure 7, Table S1). 

**Figure 8:**
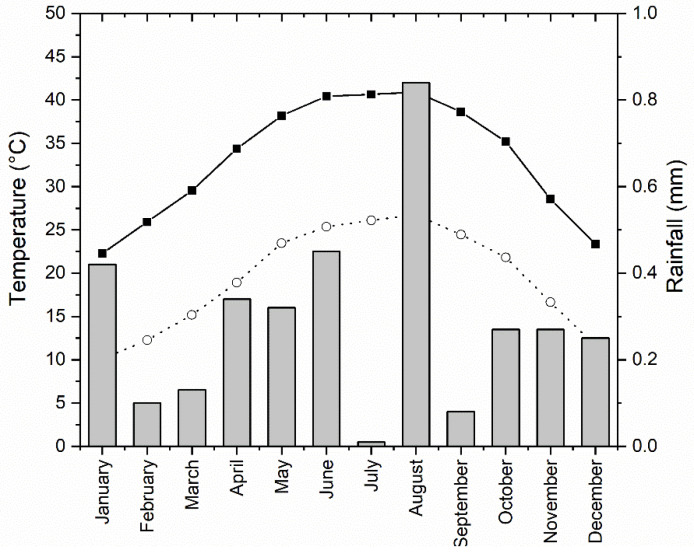
Monthly averages of rainfall (mm; bars), and maximum (black circles) and minimum (open circles) monthly air temperature (°C) at Aswan through the last ten years (2009–2019)

The study area lies within a hyper-arid zone characterized by high daily temperature and atmospheric humidity [61]. The temperature is regular in its seasonality, with 26.1 °C average annual temperature. Winter months are cold, with 10.09 °C minimum average air temperature in January, whereas the summer months are hot, with 40.91 °C maximum average air temperature in August. The rainfall is sporadic and unpredictable, with an average annual precipitation of 0.29 mm.year^−1^, with a ten-year monthly mean fluctuating between 0.01 mm in July and 0.84 mm in August (Raft Meteorological Station, 2 km from Aswan High Dam; 23°58′18″ N, 32°51′01″ E) (Figure 8).

### 4.2. Water Fluctuation in Aswan Reservoir 

The Aswan High Dam (AHD) operation has influenced the flow rate and flow level downstream to the Aswan Old Dam (AOD) throughout the year. Historical records show a remarkable reduction in the maximum flow rate after the construction of the AHD. This variation on water flow discharge throughout the year extends between June and August. During the period of maximum flow rate, release can reach up to 4.5 times the minimum flow rate. The fluctuation in the flow rate is reflected in the water level downstream of the AOD. The flow rate had dropped sharply after the operation of the AHD and suspended sediment transport as well (Figure S4).

### 4.3. Vegetation Sampling and Species Identification

Vegetation was sampled in 11 localities along the study area, divided into three zones (Eastern bank, Middle islands, and Western bank), representing a range of land uses from inhabited regions with agriculture and uninhabited areas with natural vegetation. The sampling was conducted in different seasons over the period from September 2017 to March 2020. A total of 255 plots (each of 5 × 5 m) located randomly within 27 permanently visited stands were selected in the study area (Table S1) based on the plant cover according to the Reléve method [62]. 

The surveyed taxa were identified and named according to the available literature [22,23,24,25], and names were updated [63]. Life-form categories along with life span were identified [43,64]. Phytogeographical affinities of the recorded taxa were defined [65,66,67]. Voucher specimens were deposited in the ASW herbarium, Egypt (herbarium acronyms following [68]).

### 4.4. Soil Sampling and Analyses

Three soil samples were collected at different profiles (0–20 and 20–50 cm) from randomly selected points per plot. The samples were collected twice, in summer (dry season) and winter (wet season). The three samples from the same site were mixed into one composite piece for analysis for a given profile. The air-dried soil samples were homogenized and passed through a 2 mm sieve to remove gravel. Soil texture was determined using the pipette method, and the percentages of sand, silt, and clay were calculated [69]. The total organic matter was determined by computing the weight loss after the ignition at 600 °C [70]. Soil cations elements (Ca, Mg, K, and Na) were determined. Na and K were determined by flame emission photometry according to the method proposed by [71]. Ca and Mg were determined volumetrically by the versine titration method [72]. Analyses of soil anions included the determination of total carbonates (CO_3_), bicarbonates (HCO_3_) by titration using HCl 0.01N [73], chlorides (Cl) determined volumetrically by precipitation as AgCl [74], sulfates (SO_4_) were estimated by turbidimetry as BaSO_4_ using colorimeter [75], dissolved inorganic orthophosphates were determined colorimetrically as phospho-molybdate according to [76], and nitrates (NO_3_) were determined spectrophotometrically by sodium salicylate [77]. 

### 4.5. Data Analysis

Correlation analysis (similarity and dissimilarity [78]) between each pair of sites within the study area was carried out using Corrplot in R project V.3.2.2. [79] based on the Euclidean distance measure and using the unweighted pair group method using the arithmetic averages (UPGMA) clustering algorithm [80]. Multivariate analyses were applied to evaluate the Aswan Reservoir vegetation using classification and ordination techniques from presence/absence data matrices. The floristic data matrix was then classified into vegetation groups by the two-way indicator species analysis (TWINSPAN) using the default settings of the computer program CAP for Windows (community analysis package, version 1.2) [81]. With the minimum variance as an algorithm, a dendrogram was elaborated. The species were clustered based on the samples’ classification, following a divisive hierarchical clustering of sites. Species richness (alpha-diversity) within each TWINSPAN vegetation group was calculated as the average number of species per site, and species turnover (beta-diversity) as the ratio between the total species recorded in a certain vegetation cluster and its alpha diversity [82,83]. Detrended correspondence analysis (DCA), an indirect ordination technique, was used to describe changes in the vegetation along the environmental gradients such as altitude, water zone proximity, and soil variables [84]. Canonical correspondence analysis (CCA) was performed to depict the correlations between vegetation groups and environmental data by the CANOCO program version 4.5, using species cover, stands, and soil as variables [85]. Pearson’s linear correlation coefficient [*r*] was used to assess the relationship of the measured edaphic variables among the vegetation assemblages, using SPSS program Version-20 [86].

## 5. Conclusions

This investigation provided recent information on habitat variation and vegetation structure in relation to edaphic factors in the Aswan Reservoir area, a lake between two dams in upper Egypt. Moreover, it highlighted the importance of edaphic variables (concentration of Ca, Na, Cl, HCO_3_, CO_3_, SO_3_, PO_4_, pH, total dissolved salts, and organic matter content), water fluctuations, topographic diversity, and prolonged human interference (farming and urbanization) in explaining plant diversity in the study area. Four distinct microhabitats were depicted: cultivated land, island shore, bank slope, and shoreline microhabitats. The key elements of the edaphic factors for each microhabitat may be helpful in the conservation of the Aswan reservoir ecosystem. Thus, there is a need for incorporating plant community composition, seasonality, and different sampling methods into future studies aimed at assessing the impact of dams on riparian vegetation. The study has significant conservation implications, and it establishes a baseline of ecological attributes and characteristics, such as species richness and composition that make up the plant community. Finally, the authors recommend that the Aswan reservoir area should be under management and protection because the documented severe uncontrolled human activities and urbanization would continue leading to an irreparable loss of plant diversity. 

## Figures and Tables

**Table 1:** Pearson correlation between plant species richness in the surveyed sites within the Aswan reservoir area. Sh: El Shallal area, Aw: Awad island, Ph: Philae Port, He: Heisa island, Bi: Bigga Island, Ag: Agilkia island, Bu: Bute El-Hasaya, Ti: Tingar, My: El Mahgar Valley, Mz: Maezana Belal, Hi: High Dam Colony.

Correlations											
	Sh	Bu	Mz	Hi	Ph	My	Ti	Aw	He	Bi	Ag
Sh											
Bu	−0.23										
Mz	0.08	0.58									
Hi	0.21	0.81 **	0.31								
Ph	−0.57	0.63 *	0.61 *	0.21							
My	−0.37	−0.31	−0.23	−0.51	−0.13						
Ti	0.61 *	−0.42	−0.25	0.12	−0.47	−0.16					
Aw	−0.70 *	0.43	0.38	−0.02	0.67 *	0.55	−0.49				
He	−0.69 *	0.39	0.34	−0.09	0.61 *	0.60	−0.56	0.90 **			
Bi	−0.22	0.04	0.06	−0.30	0.07	0.47	−0.62 *	0.27	0.41		
Ag	−0.11	−0.01	0.02	−0.31	−0.01	0.42	−0.59	0.16	0.31	0.91 **	

* Correlation is significant at the 0.05 level (2-tailed). ** Correlation is significant at the 0.01 level (2-tailed).

**Table 2:** The mean ± standard deviation of the soil variables of stands supporting the 10 clusters resulting after the application of TWINSPAN classification. A: 0–25 cm depth, B: 25–50 cm depth.

Edaphic Factors	I	II	III	IV	V	VI	VII	VIII	IX	X
PH	A: 7.43 ± 0.055	8.27 ± 0.053	8.09 ± 0.297	7.18 ± 0.04	7.45 ± 0	7.37 ± 0.207	7.77 ± 0.21	7.493 ± 0.159	7.67 ± 0.192	7.56 ± 0
	B: 7.45 ± 0.08	8.367 ± 0.071	8.117 ± 0.290	7.41 ± 0.02	7.415 ± 0.005	7.447 ± 0.106	7.675 ± 0.335	7.517 ± 0.061	7.33 ± 0.38	7.813 ± 0.058
TDS (mg/L)	A: 0.035 ± 0.005	0.02 ± 0.01	0.023 ± 0.006	0.015 ± 0.005	0.02 ± 0	0.023 ± 0.006	0.03 ± 0.01	0.023 ± 0.016	0.017 ± 0.01	0.05 ± 0
	B: 0.035 ± 0.005	0.02 ± 0.01	0.02 ± 0	0.02 ± 0.01	0.015 ± 0.005	0.023 ± 0.012	0.015 ± 0.005	0.02 ± 0.01	0.027 ± 0.01	0.037 ± 0.006
EC (µs/cm)	A: 122.5 ± 4.5	157 ± 49.1	159 ± 49.7	126 ± 1	103.5 ± 1.5	124.3 ± 21	117 ± 6	132.67 ± 2.89	101.3 ± 5.03	205 ± 0
	B: 99.95 ± 11.05	152.7 ± 55.4	147.3 ± 48.3	135 ± 14	106.5 ± 1.5	125.0 ± 21.1	113.67 ± 4.93	115.33 ± 5.69	88.3 ± 15.01	191.67 ± 2.89
Organic matter %	A: 1.04 ± 0.117	3.57 ± 1.29	1.215 ± 0.128	0.978 ± 0.006	0.637 ± 0.045	0.864 ± 0.1344	0.573 ± 0.181	0.749 ± 0.258	0.647 ± 0.23	0.869 ± 0
	B:0.035 ± 0.005	3.682 ± 1.429	1.445 ± 0.571	0.915 ± 0.039	0.682 ± 0.254	0.823 ± 0.11	0.599 ± 0.071	0.874 ± 0.282	0.704 ± 0.2	0.937 ± 0.02
Na^+^ (mg/g)	A: 16.02 ± 5.85	12.2 ± 1.77	26.31 ± 10.02	19.44 ± 0.999	29.9 ± 1.23	19.05 ± 5.18	8.603 ± 0.072	9.71 ± 2.48	10.96 ± 3.1	11.27 ± 0
	B: 13.44 ± 3.9	10.14 ± 2.45	23.57 ± 9.1	19.064 ± 1.72	25.015 ± 0.519	18.07 ± 5.59	8.141 ± 0.274	8.2 ± 1.94	11.10 ± 2.12	9.59 ± 0.403
K^+^ (mg/g)	A: 1.31 ± 0.566	0.79 ± 0.196	1.296 ± 0.379	1.036 ± 0.119	1.779 ± 0.0924	1.499 ± 0.382	1.27 ± 0.377	0.631 ± 0.21	1.27 ± 0.798	1.602 ± 0
	B: 1.16 ± 0.62	1.004 ± 0.233	1.057 ± 0.387	0.905 ± 0.096	1.4475 ± 0.0385	1.532 ± 0.492	0.589 ± 0.012	0.547 ± 0.121	1.17 ± 0.019	0.57 ± 0.004
Ca^2+^ (mg/g)	A:0.6 ± 0.1	0.833 ± 0.088	0.6 ± 0.033	0.633 ± 0	0.733 ± 0	0.622 ± 0.051	0.483 ± 0.05	0.624 ± 0.181	0.567 ± 0.1	0.467 ± 0
	B: 0.383 ± 0.05	0.656 ± 0.084	0.544 ± 0.157	0.533 ± 0.033	0.567 ± 0.167	0.444 ± 0.084	0.383 ± 0.05	0.518 ± 0.13	0.433 ± 0.1	0.264 ± 0.02
Mg^2+^ (mg/g)	A: 0.4 ± 0.08	0.347 ± 0.18	0.4 ± 0.08	0.42 ± 0.02	0.34 ± 0.06	0.413 ± 0.046	0.52 ± 0.04	0.427 ± 0.201	0.36 ± 0.069	0.44 ± 0
	B: 0.24 ± 0.04	0.213 ± 0.083	0.32 ± 0.183	0.3 ± 0.06	0.54 ± 0.34	0.24 ± 0.106	0.38 ± 0.1	0.334 ± 0.151	0.4 ± 0	0.367 ± 0.012
Cl^−^(mg/g)	A: 0.095 ± 0	0.063 ± 0.025	0.059 ± 0.021	0.065 ± 0.006	0.071 ± 0.024	0.063 ± 0.018	0.053 ± 0.006	0.079 ± 0.027	0.059 ± 0	0.047 ± 0
	B: 0.059 ± 0.012	0.059 ± 0.012	0.051 ± 0.014	0.065 ± 0.018	0.077 ± 0.005	0.071 ± 0.024	0.041 ± 0.006	0.055 ± 0.014	0.055 ± 0.03	0.054 ± 0.012
CO_3_^2−^ (mg/g)	A: 2.56 ± 0.019	1.44 ± 0.312	2.58 ± 0.287	1.92 ± 0.166	1.863 ± 0.1	1.757 ± 0.434	1.798 ± 0.14	2.078 ± 0.317	2.592 ± 0.45	2.438 ± 0
	B: 1.99 ± 0.337	1.716 ± 0.268	1.809 ± 0.151	1.75 ± 0.079	1.62 ± 0.279	1.267 ± 0.439	1.53 ± 0.143	1.66 ± 0.161	1.474 ± 0.42	2.04 ± 0.006
PO_4_^2−^ (mg/g)	A: 0.014 ± 0	0.033 ± 0.004	0.049 ± 0.026	0.036 ± 0.003	0.013 ± 0	0.048 ± 0.035	0.043 ± 0.002	0.016 ± 0.003	0.023 ± 0.01	0.031 ± 0
	B: 0.008 ± 0.005	0.028 ± 0.006	0.04 ± 0.026	0.026 ± 0.005	0.029 ± 0	0.045 ± 0.031	0.023 ± 0.014	0.015 ± 0.004	0.027 ± 0.01	0.04 ± 0.003
NO_3_^−^ (mg/g)	A: 0.19 ± 0.025	0.166 ± 0.005	0.196 ± 0.024	0.167 ± 0.041	0.139 ± 0.0137	0.143 ± 0.016	0.163 ± 0.008	0.196 ± 0.045	0.127 ± 0.05	0.165 ± 0
	B: 0.171 ± 0.015	0.162 ± 0.049	0.161 ± 0.018	0.157 ± 0.029	0.14 ± 0.007	0.142 ± 0.021	0.119 ± 0.011	0.146 ± 0.02	0.105 ± 0.02	0.138 ± 0.03
SO_4_^2−^ (mg/g)	A: 0.091 ± 0.025	0.034 ± 0.027	0.225 ± 0.045	0.268 ± 0.04	0.125 ± 0	0.167 ± 0.057	0.068 ± 0.003	0.0527 ± 0.01	0.059 ± 0.03	0.164 ± 0
	B: 0.091 ± 0.02	0.0341 ± 0.03	0.2252 ± 0.045	0.243 ± 0.066	0.125 ± 0	0.167 ± 0.057	0.068 ± 0.003	0.053 ± 0.01	0.041 ± 0.03	0.15 ± 0.015
Soil texture	Sand loamy sand	sandy clay loam clay loam	clay loam	sand	sand	sand	sand	sand	sandy loam	sand

## Data Availability

The data that support the findings of this study are available from the corresponding author upon reasonable request.

## Supplementary Materials

The following are available online at https://www.mdpi.com/article/10.3390/plants10122712/s1, Figure S1: Histogram shows the species’ numbers in each of the 46 families of angiosperms surveyed in the Aswan Reservoir area, upper Egypt., Figure S2: Soil texture and organic matter content at different soil depths (0–25 and 25–50 cm) concerning the TWINSPAN vegetation groups in the studied area., Figure S3: The concentration of cations and anions (mg.g^−1^) in the TWINSPAN vegetation groups., Figure S4: The fluctuation of average daily inflows at the Aswan Reservoir (October 2017–December 2018), Egypt., Table S1: Locations of the studied areas in Aswan Reservoir with their coordinates, human activity, and number of stands, Table S2: Synoptic table of species composition of the ten vegetation groups (I–X) identified after application of TWINSPAN to the vegetation data of 170 taxa in 27 stands along the Aswan reservoir area along with plant life forms, chorotypes, and presence value.
