# Supplementary material for: Vegetation Analysis and Environmental Relationships of Riverain Plants in the Aswan Reservoir, Egypt
Source: Plants (Basel). 2021 Dec 10;10(12):2712. doi: 10.3390/plants10122712 (PMC8707900; doi:10.3390/plants10122712)
Supplement: Supplementary file 1 [file plants-10-02712-s001.zip › Table S1.pdf]

Table S1. Locations of the studied areas in Aswan Reservoir with their coordinates, human activity, number of stands, and number of plots within each stand.

Locations	Human activity	No. of Stands	No. of plots (quadrates)	Coordinates	
				Latitude (N)	Longitude (E)
El Shallal	Inhabited	10	100	24°01'47.55"	32°53'52.22"
Bute El-Hasaya	Uninhabited	1	10	24°00'54.29"	32°53'30.81"
Maezana Belal	Uninhabited	1	10	24°00'30.17"	32°53'21.88"
High Dam Colony	Inhabited	3	20	23°59'03.69"	32°52'55.01"
Philae Port	Uninhabited	1	10	24°02'02.03"	32°53'09.15"
El Mahgar Valley	Uninhabited	3	30	24°00'17.62"	32°52'14.72"
Tingar	Inhabited	2	30	24°59'37.85"	32°52'12.28"
Awad	Inhabited	1	5	24°01'43.26"	32°52'24.80"
Heisa	Inhabited	2	10	24°00'19.88"	32°52'32.08"
Bigga	Inhabited	1	10	24°01'15.69"	32°52'24.80"
Agilkia	Uninhabited	2	20	24°01'17.31"	32°53'22.44"
**Total**		**27**	**255**		
