# Supplementary material for: Vegetation Analysis and Environmental Relationships of Riverain Plants in the Aswan Reservoir, Egypt
Source: Plants (Basel). 2021 Dec 10;10(12):2712. doi: 10.3390/plants10122712 (PMC8707900; doi:10.3390/plants10122712)
Supplement: Supplementary file 1 [file plants-10-02712-s001.zip › Table S2.pdf]

Table S2. Synoptic table of species composition of the ten vegetation groups (I–X) identified after application of TWINSPAN to the vegetation data of 170 taxa in 27 stands along Aswan reservoir area along with plant life forms, chorotypes, and presence value.

Species	Life form	Chorotype	Vegetation groups										P%
			I	II	III	IV	V	VI	VII	VIII	IX	X	
			2	3	4	2	2	3	2	4	4	1	
**Species present in ten clusters**													
Cynodon dactylon (L.) Pers.	Ge	COSM	100	100	100	100	100	100	100	100	100	100	100
Euphorbia hirta L.	Th	NEO	50	66.67	75	100	100	100	100	100	100	100	92.59
Imperata cylindrica (L.) P.Beauv.	Ge	IT + ME+ SS + SZ	100	100	50	100	100	66.6	100	100	100	100	92.59
*Leptadenia arborea (*Forssk.) Schweinf.	Ph	SA + SZ	100	100	100	100	100	100	100	75	75	100	96.30
Tephrosia purpurea subsp. *apollinea* (Delile) Hosni & El-Karemy	Ch	IT + SS + SZ	50	33.33	100	100	100	66.6	100	75	75	100	77.78
**Species present in nine clusters**													
Cuscuta pedicellata Ledeb.	Pa	COSM	50	66.67	75	100	100	100	100	50	25	0	66.67
Phoenix dactylifera L.	Ph	IT + SA	100	100	100	100	100	66.67	100	50	100	0	85.19
Phragmites australis (Cav.) Trin. ex Steud.	GH	COSM	100	0	75	100	100	66.67	100	100	50	100	85.19
Pluchea dioscoridis (L.) DC.	Ph	SS + SZ	100	100	25	100	50	66.67	50	100	25	0	62.96
Psidium guajava L.	Ph	NEO	50	0	75	50	100	100	100	100	100	100	81.48
Pulicaria undulata (L.) C.A.Mey.	Ch	IT + ME + SS + SZ	50	66.67	50	50	100	66.67	0	75	25	100	55.56
Sesbania sesban (L.) Merr.	Ph	SA + SZ	50	0	75	100	100	100	100	100	100	100	81.48
**Species present in eight clusters**													
Calotropis procera (Aiton) W.T.Aiton	Ph	SA + SZ	100	100	75	100	100	33.33	50	75	0	0	62.96
Dichanthium annulatum (Forssk.) Stapf	Hem	PAN	100	100	100	100	100	100	0	100	25	0	77.78
Euphorbia peplus L.	Th	ES + IT + ME + SS + SZ	50	0	50	50	100	66.67	100	75	25	0	51.85
Lantana camara L.	Ph	NEO	100	100	75	0	100	100	100	25	50	0	66.67
Setaria geminata (Forssk.) Veldkamp	Ge	ME + SS + SZ	50	33.33	50	50	100	66.67	0	75	0	100	48.15
Phyla nodiflora (L.) Greene	Hem	PAN	0	0	50	50	100	100	50	50	25	100	48.15
Portulaca oleracea L.	Th	IT + ME + SA + SZ	0	33.33	75	50	100	66.67	50	100	0	100	55.56
Solanum nigrum L.	Th	COSM	0	33.33	50	50	100	33.33	100	25	0	100	44.44
Tamarix nilotica (Ehrenb.) Bunge	Ph	ME + SA + SZ	100	0	50	100	100	66.67	100	75	100	0	70.37
**Species present in seven clusters**													
Ageratum conyzoides L.	Th	NEO	0	0	0	100	100	100	100	100	75	100	62.96
Ceratophyllum demersum L.	Hy	COSM	0	0	0	50	100	100	100	100	75	100	59.26
Leucaena leucocephala (Lam.) de Wit	Ph	NEO	50	33.33	25	50	100	66.67	0	0	25	0	33.33
Paspalum distichum L.	Ge	NEO	50	33.33	75	50	100	66.67	0	25	0	0	37.04
Caroxylon imbricatum (Forssk.) Moq.	Ch	IT + ME + SS + SZ	100	33.33	25	100	0	33.33	0	25	25	0	33.33
Stuckenia pectinata (L.) Börner.	Hy	COSM	0	0	0	100	100	100	100	25	75	100	51.85

Symphyotrichum subulatum var. *squamatum* (Spreng.) S.D.Sundb.	Ch	NEO	0	0	0	100	100	100	100	100	25	100	55.56
Trigonella glabra Thunb. subsp. *glabra*	Th	ME + SS + SZ	0	33.33	50	50	50	33.33	100	75	0	0	40.74
Veronica anagallis-aquatica L.	GH	COSM	0	0	0	50	100	100	100	50	25	100	44.44
Ziziphus spina-christi (L.) Desf.	Ph	IT + ME + SA + SZ	50	33.33	50	100	50	33.33	0	25	0	0	33.33
**Species present in six clusters**													
Abutilon pannosum (G.Forst.) Schltdl.	Ch	IT + ME + SS + SZ	0	66.67	50	100	100	33.33	0	50	0	0	40.74
Alhagi maurorum subsp. *graecorum* (Boiss.) Awmack & Lock	Ch	IT + ME + SA + SZ	50	33.33	75	100	100	33.33	0	0	0	0	37.04
Lysimachia arvensis (L.) U.Manns & Anderb.	Th	ES + IT + ME + SS + SZ	0	33.33	50	0	100	66.67	100	50	0	0	40.74
Cyperus laevigatus L.	Ge	PAN	0	0	0	50	50	66.67	50	25	0	100	25.93
Cyperus longus L.	He	IT + ME + SS + SZ	0	0	0	0	100	66.67	100	100	25	100	44.44
Eleocharis geniculata (L.) Roem. & Schult.	He	PAN	0	0	0	50	100	66.67	100	50	0	100	37.04
Helichrysum luteoalbum (L.) Rchb.	Th	COSM	0	0	25	50	50	0	50	25	0	100	22.22
Lepidium didymum L.	Th	NEO	0	0	0	50	100	100	50	50	0	100	37.04
Melilotus indicus (L.) All.	Th	IT + ME + SS	0	66.67	25	0	100	33.33	100	25	0	0	33.33
Polypogon monspeliensis (L.) Desf.	Th	IT + ME + SS + SZ + SJ	50	33.33	25	50	50	0	0	25	0	0	22.22
Potamogeton crispus L.	Hy	PAL	0	0	0	0	100	100	50	75	50	100	44.44
Rorippa palustris (L.) Besser	Th	COSM	0	0	0	100	100	33.33	100	100	0	100	44.44
**Species present in five clusters**													
Vachellia farnesiana (L.) Wight & Arn.	Ph	NEO	0	0	25	0	100	33.33	100	0	25	0	25.93
Alternanthera sessilis (L.) R.Br. ex DC.	Th	PAN	0	0	0	100	100	100	0	100	0	100	44.44
Amaranthus blitum subsp. *oleraceus* (L.) Costea	Th	NEO	50	0	0	100	100	33.33	50	0	0	0	25.93
Chenopodium album L.	Th	COSM	0	33.33	50	0	100	0	50	25	0	0	25.93
Chenopodiastrum murale (L.) S.Fuentes, Uotila & Borsch	Th	COSM	0	33.33	50	50	0	0	0	25	25	0	22.22
Cyperus rotundus L.	Ge	COSM	50	33.33	25	0	50	0	0	50	0	0	22.22
Dalbergia sissoo Roxb. ex DC.	Ph	IT + SZ	0	33.33	50	50	0	0	50	75	0	0	29.
Digitaria sanguinalis (L.) Scop.	Th	IT + ME + SS + SZ + SJ	0	33.33	25	50	50	0	0	100	0	0	29.63
Echinochloa colona (L.) Link	Th	PAL	50	33.33	25	0	0	33.33	0	25	0	0	18.52
Hyphaene thebaica (L.) Mart.	Ph	SA + SZ	50	0	50	100	50	33.33	0	0	0	0	25.93
Malva parviflora L.	Th	IT + ME + SS + SZ	50	33.33	50	0	50	0	0	25	0	0	18.52
Myriophyllum spicatum L.	Hy	COSM	0	0	0	0	100	66.67	50	100	25	0	37.04
Nerium oleander L.	Ph	IT + ME + SS + SZ	50	66.67	50	0	0	33.33	0	25	0	0	25.93
Persicaria decipiens (R.Br.) K.L.Wilson	GH	IT + ME + SS + SZ	0	0	0	50	100	100	100	50	0	0	37.04
*Persicaria senegalensis (*Meisn.) Soják	GH	IT + ME + SA + SZ	0	0	0	50	50	66.67	50	75	0	0	29.63
Physalis angulata L.	Th	NEO	0	0	0	100	100	100	100	100	0	0	48.15
Potamogeton perfoliatus L.	Hy	COSM	0	0	0	0	100	33.33	100	50	50	0	33.33
Senna occidentalis (L.) Link	Ch	NEO	0	0	0	50	100	33.33	0	25	0	100	22.22

Trifolium resupinatum L.	Th	IT + ME + SS	0	0	0	100	100	33.33	50	25	0	0	25.93
**Species present in four clusters**													
Vachellia seyal (Delile) P.J.H.Hurter	Ph	SA + SZ	0	0	0	100	100	0	50	0	50	0	25.93
Aerva javanica (Burm.f.) Juss. ex Schult.	Th	PAL	100	66.67	0	50	0	33.33	0	0	0	0	22.22
Amaranthus spinosus L.	Th	NEO	0	0	25	50	0	33.33	0	50	0	0	18.52
Ammannia baccifera L.	Th	PAN	0	0	0	0	100	66.67	50	25	0	0	2
Argemone mexicana L.	Th	NEO	50	0	0	50	100	0	50	0	0	0	18.52
Bougainvillea glabra Choisy	Ph	NEO	50	66.67	25	50	0	0	0	0	0	0	18.52
Corchorus olitorius L.	Th	PAL	100	66.67	50	0	0	0	0	75	0	0	33.33
Cyclospermum leptophyllum (Pers.) Sprague	Th	NEO	0	0	0	0	50	66.67	100	50	0	0	25.93
Dactyloctenium aegyptium (L.) Willd.	Th	PAL	0	33.33	50	50	0	0	0	25	0	0	18.52
Desmostachya bipinnata (L.) Stapf	Ge	PAL	50	100	0	50	0	33.33	0	0	0	0	22.22
Eleocharis parvula (Roem. & Schult.) Link ex Bluff, Nees & Schauer	Hem	COSM	0	0	0	0	100	33.33	50	25	0	0	18.52
Eragrostis cilianensis (All.) Vignolo ex Janch.	Th	IT + ME + SS + SZ + SJ	0	100	25	0	0	33.33	0	100	0	0	33.33
Glinus lotoides L.	Th	COSM	0	0	50	50	100	66.67	0	0	0	0	25.93
Lepidium coronopus (L.) Al-Shehbaz	Th	ER-SR + IT + ME + SA	0	0	0	100	100	100	0	100	0	0	40.74
Plantago major L.	Hem	COSM	0	0	25	50	100	100	0	0	0	0	25.93
Poa annua L.	Th	ER-SR + IT + ME + SS + SZ + SJ	0	33.33	50	50	100	0	0	0	0	0	22.22
Rumex dentatus L.	Th	PAL	50	0	0	100	0	66.67	0	25	0	0	22.22
Sonchus oleraceus L.	Th	ER-SR + IT + ME + SS + SZ	50	0	75	0	0	0	50	25	0	0	22.22
Syzygium cumini (L.) Skeels	Ph	PAN	0	0	50	50	0	33.33	0	50	0	0	22.22
Typha domingensis Pers.	He	PAN	50	0	0	50	50	0	0	25	0	0	14.81
**Species present in three clusters**													
Vachellia nilotica (L.) P.J.H.Hurter & Mabb.	Ph	IT + SS + SZ	0	0	0	100	0	0	0	25	25	0	14.81
Arundo donax L.	Hy	IT + ME + SZ	0	66.67	25	0	50	0	0	0	0	0	14.81
Bidens pilosa L.	Th	NEO	0	0	75	50	0	0	0	25	0	0	18.52
Casuarina equisetifolia L.	Ph	PAN	50	66.67	25	0	0	0	0	0	0	0	14.81
Cenchrus biflorus Roxb.	Th	IT + SS	0	33.33	75	0	50	0	0	0	0	0	18.52
Chloris pycnothrix Trin.	Th	PAN		0	0	0	100	0	0	50	0	0	14.81
Citrullus colocynthis (L.) Schrad.	Hem	IT + ME + SS + SZ	50	0	50	0	0	0	0	0	50	0	18.52
Datura innoxia Mill.	Th	NEO	0	66.67	25	0	50	0	0	0	0	0	14.81
Rumex spinosus L.	Th	IT + ME + SA + SZ	0	0	0	50	0	100	50	0	0	0	18.52
Eruca vesicaria (L.) Cav.	Th	IT + ME + SS + SZ	0	33.33	75	0	0	33.33	0	0	0	0	18.52
Euphorbia forskaolii J.Gay	Th	IT + ME + SS + SZ	0	33.33	25	50	0	0	0	0	0	0	11.11
Euphorbia heterophylla L.	Th	NEO	0	66.67	25	0	0	0	0	25	0	0	14.81
Fimbristylis bisumbellata (Forssk.) Bubani	Th	COSM	0	0	0	50	0	33.33	0	25	0	0	11.11

Forsskaolea tenacissima L.	Hem	IT + ME + SS + SZ	50	66.67	0	50	0	0	0	0	0	0	14.81
Ipomoea cairica (L.) Sweet	Hem	PAL	50	33.33	75	0	0	0	0	0	0	0	25.93
Khaya senegalensis (Desv.) A.Juss.	Ph	SZ	50	66.67	25	0	0	0	0	0	0	0	14.81
Lactuca sativa L.	Th	IT	0	33.33	50	0	50	0	0	0	0	0	14.81
Lactuca serriola L.	Th	ER-SR + IT + ME + SS + SZ	0	33.33	75	0	100	0	0	0	0	0	22.22
Lotus arabicus Sol. ex L.	Th	SS + SZ	0	0	25	50	100	0	0	0	0	0	14.81
Oxalis corniculata L.	GH	NEO	0	33.33	75	0	0	0	0	75	0	0	25.93
Polygonum aviculare L.	Th	COSM	0	0	0	0	100	66.67	0	50	0	0	22.22
Senna didymobotrya (Fresen.) H.S.Irwin & Barneby	Ph	SZ	0	0	0	50	100	0	0	25	0	0	14.81
Senna italica Mill.	Ch	IT + ME + SS + SZ	0	33.33	25	0	0	0	0	25	0	0	11.11
Setaria viridis (L.) P.Beauv.	Th	COSM	0	33.33	75	0	0	0	0	25	0	0	18.52
Trianthema portulacastrum L.	Th	PAN	0	100	25	0	100	0	0	0	0	0	22.22
Tribulus terrestris L.	Th	ER-SR + IT + ME + SS + SZ + SJ	0	0	25	100	50	0	0	0	0	0	14.81
**Species present in two clusters**													
Vachellia tortilis subsp. *raddiana* (Savi) Kyal. & Boatwr.	Ph	IT + ME + SS + SZ	0	0	0	100	0	0	0	0	25	0	11.11
Ammi majus L.	Th	IT + ME + SS + SZ	0	33.33	25	0	0	0	0	0	0	0	7.41
Avena fatua L.	Th	COSM	0	33.33	50	0	0	0	0	0	0	0	11.11
Balanites aegyptiaca (L.) Delile	Ph	IT + ME + SS + SZ	0	0	25	0	50	0	0	0	0	0	7.41
Pseudoconyza viscosa (Mill.) D'Arcy	Th	PAN	0	0	50	0	0	33.33	0	0	0	0	11.11
Coincya tournefortii (Gouan) Alcaraz, T.E.Díaz, Rivas Mart. & Sánchez-Gómez	Th	IT + ME + SS	0	66.67	50	0	0	0	0	0	0	0	14.81
Cardiospermum halicacabum L.	Th	PAN	0	33.33	25	0	0	0	0	0	0	0	7.41
Convolvulus arvensis L.	Hem	ER-SR + IT + ME + SS + SZ + SJ	0	0	50	0	50	0	0	0	0	0	11.11
Cucurbita pepo L.	Th	NEO	0	33.33	25	0	0	0	0	0	0	0	7.41
Erigeron bonariensis L.	Th	NEO	0	33.33	75	0	0	0	0	0	0	0	14.81
Eucalyptus camaldulensis Dehnh.	Ph	AUS	0	0	50	0	0	0	0	25	0	0	11.11
Haematoxylum campechianum L.	Ph	NEO	0	0	0	0	50	0	50	0	0	0	7.41
Ipomoea carnea Jacq.	Ph	NEO	0	66.67	50	0	0	0	0	0	0	0	14.81
Ipomoea eriocarpa R. Br.	Th	PAN	0	66.67	25	0	0	0	0	0	0	0	11.11
Lawsonia inermis L.	Ph	SA + SZ	0	33.33	25	0	0	0	0	0	0	0	7.41
Lolium perenne L.	Th	ER-SR + IT + ME + SA	0	0	50	0	0	0	0	25	0	0	11.11
Panicum coloratum L.	Ge	SA + SZ	0	33.33	25	0	0	0	0	0	0	0	0.00
Panicum repens L.	Ge	COSM	0	0	75	0	0	0	0	25	0	0	14.81
Plantago lagopus L.	Th	IT + ME + SA	0	0	0	50	0	0	0	25	0	0	7.41
Ricinus communis L.	Ph	SZ	0	66.67	50	0	0	0	0	0	0	0	14.81
Salix tetrasperma Roxb.	Ph	PAL	50	0	0	0	0	0	0	25	0	0	7.41

Senecio aegyptius L.	Th	ME + SA + SZ	0	33.33	25	0	0	0	0	0	0	0	7.41
Sida alba L.	Hem	SS + SZ	0	0	50	0	50	0	0	0	0	0	14.81
Trifolium alexandrinum L.	Th	IT + ME + SS	0	33.33	75	0	0	0	0	0	0	0	14.81
Vicia faba L.	Th	IT	0	33.33	25	0	0	0	0	0	0	0	7.41
**Species present in one cluster**													
Senegalia laeta (R.Br. ex Benth.) Seigler & Ebinger	Ph	SA + SZ	0	0	0	0	0	0	0	0	3.7	0	3.70
Adiantum capillus-veneris L.	Hem	COSM	3.7	0	0	0	0	0	0	0	0	0	3.70
Astragalus vogelii (Webb) Bornm.	Th	IT + ME + SS	0	0	0	0	3.7	0	0	0	0	0	3.70
Boerhavia repens L.	Ch	COSM	0	0	0	0	3.7	0	0	0	0	0	3.70
Bombax ceiba L.	Ph	PAN	0	0	7.4	0	0	0	0	0	0	0	7.41
Brassica nigra (L.) W.D.J.Koch	Th	COSM	0	11.11	0	0	0	0	0	0	0	0	11.11
Cajanus cajan (L.) Huth	Ph	SZ	0	0	3.7	0	0	0	0	0	0	0	3.70
Crotalaria thebaica Delile DC.	Th	SA + SZ	3.7	0	0	0	0	0	0	0	0	0	3.70
Cucumis melo L.	Th	PAN	0	0	3.7	0	0	0	0	0	0	0	3.70
Cyperus alopecuroides Rottb.	GH	PAN	0	0	0	0	0	0	0	3.7	0	0	3.70
Cyperus difformis L.	Th	COSM	0	0	0	0	0	0	0	7.4	0	0	7.41
Cyperus michelianus subsp. *pygmaeus* (Rottb.) Asch. & Graebn.	Th	PAN	0	0	0	0	0	0	0	3.7	0	0	3.70
Blumea bovei (DC.) Vatke	Ch	IT + ME + SS + SZ	3.7	0	0	0	0	0	0	0	0	0	3.70
Echium rauwolfii Delile	Th	IT + ME + SA + SZ	0	0	3.7	0	0	0	0	0	0	0	3.70
Eclipta prostrata (L.) L.	Th	NEO	3.7	0	0	0	0	0	0	0	0	0	3.70
Eleusine indica (L.) Gaertn.	Th	PAL	0	0	7.4	0	0	0	0	0	0	0	7.41
Epilobium hirsutum L.	HHe	ER-SR + IT + ME + SA + SZ + SJ	3.7	0	0	0	0	0	0	0	0	0	3.70
Zygophyllum indicum (Burm.f.) Christenh. & Byng	Ch	IT + ME + SS + SZ	0	0	0	3.7	0	0	0	0	0	0	3.70
Hibiscus sabdariffa L.	Th	SZ	0	0	11.11	0	0	0	0	0	0	0	11.11
Hibiscus sp.	Ph	PAL	0	0	0	3.7	0	0	0	0	0	0	3.70
Indigofera oblongifolia Forssk.	Ch	IT + SS + SZ	0	0	0	0	3.7	0	0	0	0	0	3.70
Juncus rigidus Desf.	GH	COSM	3.7	0	0	0	0	0	0	0	0	0	3.70
Lablab purpureus (L.) Sweet	Ch	SZ	0	0	3.7	0	0	0	0	0	0	0	3.70
Luffa aegyptiaca Mill.	Ph	PAL	0	0	0	3.7	0	0	0	0	0	0	3.70
Medicago sativa L.	Hem	IT + ME + SA	0	0	7.4	0	0	0	0	0	0	0	7.41
Mentha longifolia (L.) L.	Ch	COSM	3.7	0	0	0	0	0	0	0	0	0	3.70
Mentha pulegium L.	Th	ER-SR + IT + ME + SS + SZ	0	0	7.4	0	0	0	0	0	0	0	7.41
Moringa oleifera Lam.	Ph	SZ	0	0	7.4	0	0	0	0	0	0	0	7.41
Oldenlandia capensis L.f.	Th	IT + ME + SA + SZ	0	0	0	0	0	0	0	7.4	0	0	7.41
Oxystelma esculentum (L. f.) m.	Hem	PAN	3.7	0	0	0	0	0	0	0	0	0	3.70
*Pithecellobium dulce (*Roxb.) Benth.	Ph	NEO	0	0	0	0	0	0	0	3.7	0	0	3.70
Sesamum indicum L.	Th	SZ	0	0	7.4	0	0	0	0	0	0	0	7.41

Sorghum × drummondii (Nees ex Steud.) Millsp. & Chase	GH	SA + SZ	0	7.4	0	0	0	0	0	0	0	0	7.41
Sorghum virgatum (Hack.) Stapf	Th	IT + ME + SA + SZ	0	0	7.4	0	0	0	0	0	0	0	7.41
Tamarix aphylla (L.) H.Karst.	Ph	IT + ME + SS + SZ	3.7	0	0	0	0	0	0	0	0	0	3.70
Withania somnifera (L.) Dunal	Ch	IT + ME + SS + SZ + SJ	0	0	7.4	0	0	0	0	0	0	0	7.41
Xanthium strumarium L.	Th	NEO	0	0	0	0	0	0	0	3.7	0	0	3.70
**Species richness**			16.5	12.89	12.87	27.25	36.5	15.33	20	12.12	4.5	27	
**Species turnover**			3.15	5.58	8.08	2.82	2.35	4.63	2.55	7.34	7.78	1	

Chorotypes abbreviations: AUS: Australian, COSM: Cosmopolitan, ME: Mediterranean, NEO: Neotropical, PAL: Palaeotropical, PAN: Pantropical, ES: Euro-Siberian, IT: Irano-Turanian, SA= Saharo-Arabian, SS: Saharo-Sindian, SJ: Sino–Japonic, SZ: Sudano-Zambezian. Life form: Ch.: Chamaephyte, Ge.: Geophyte, GH: Geophyte-Helophyte, Hem.: Hemicryptophyte, Hy: Hydrophyte, HHe: Hydrophyte-Helophyte, Pa.: Parasite, Ph.: Phanerophyte, Th.: Therophyte.
