# Supplementary figures and images for: Vegetation Analysis and Environmental Relationships of Riverain Plants in the Aswan Reservoir, Egypt
Source: Plants (Basel). 2021 Dec 10;10(12):2712. doi: 10.3390/plants10122712 (PMC8707900; doi:10.3390/plants10122712)

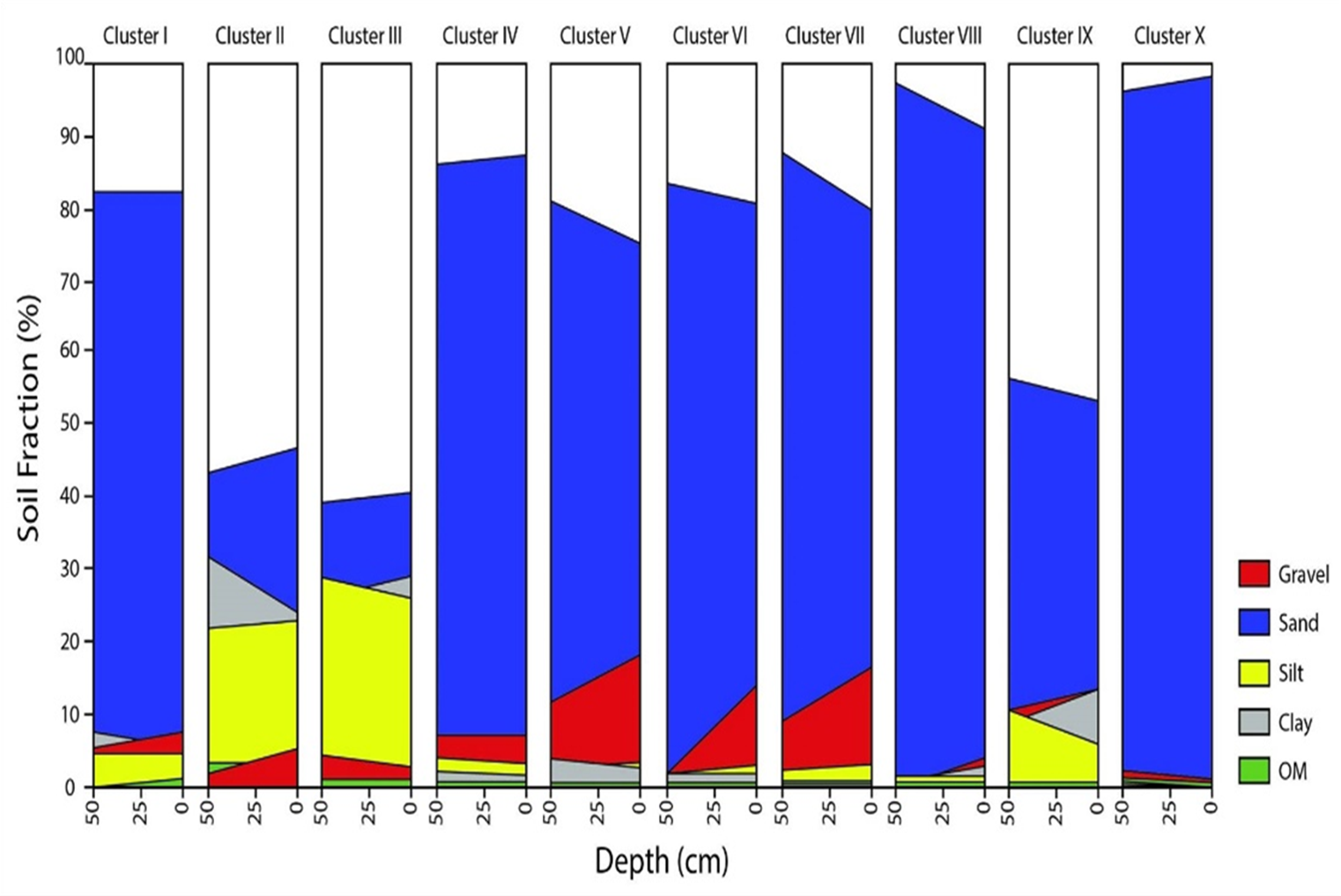

Supplement: Supplementary file 1 [file plants-10-02712-s001.zip › Figure S2.tif]

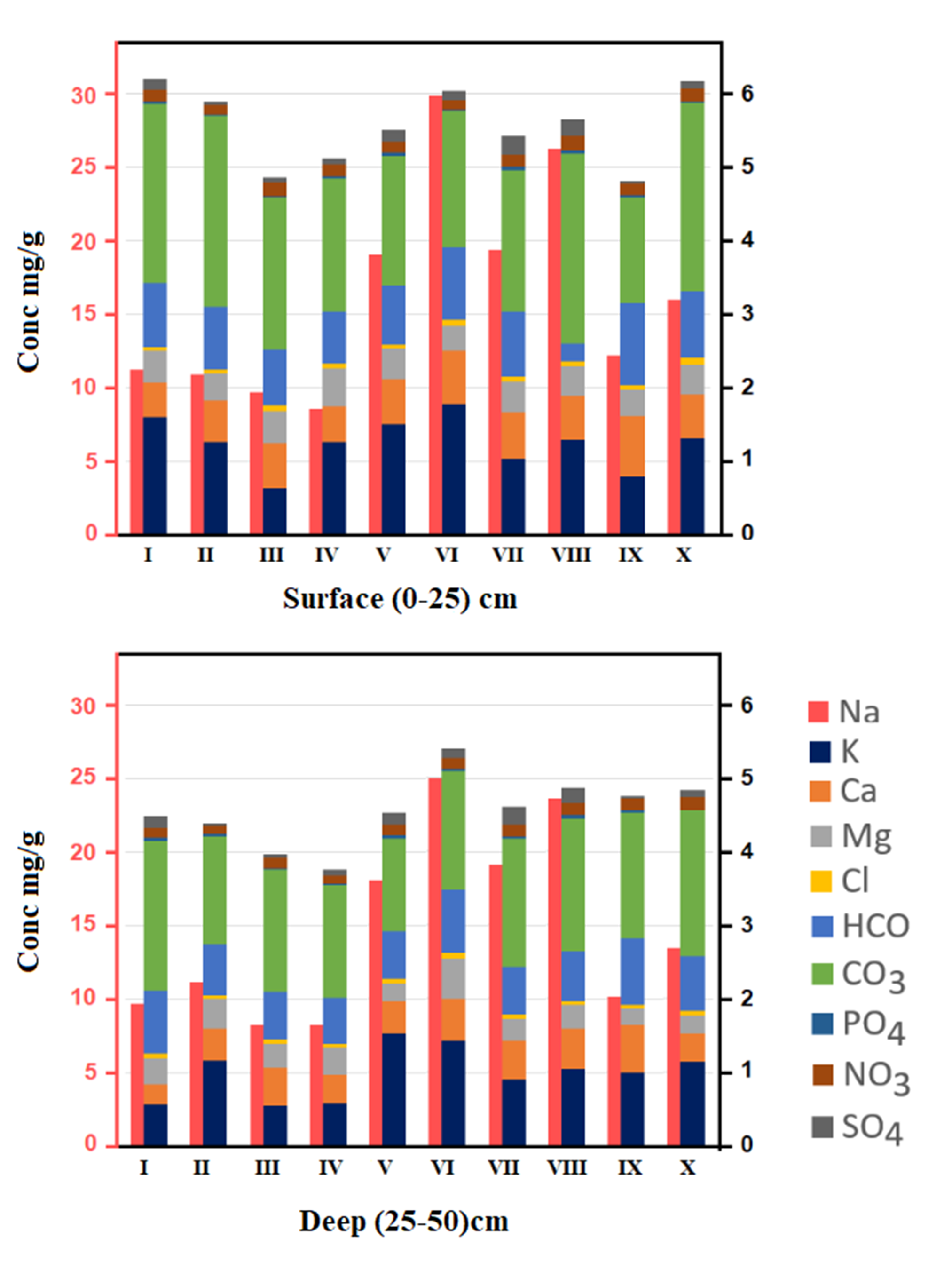

Supplement: Supplementary file 1 [file plants-10-02712-s001.zip › Figure S3.tif]

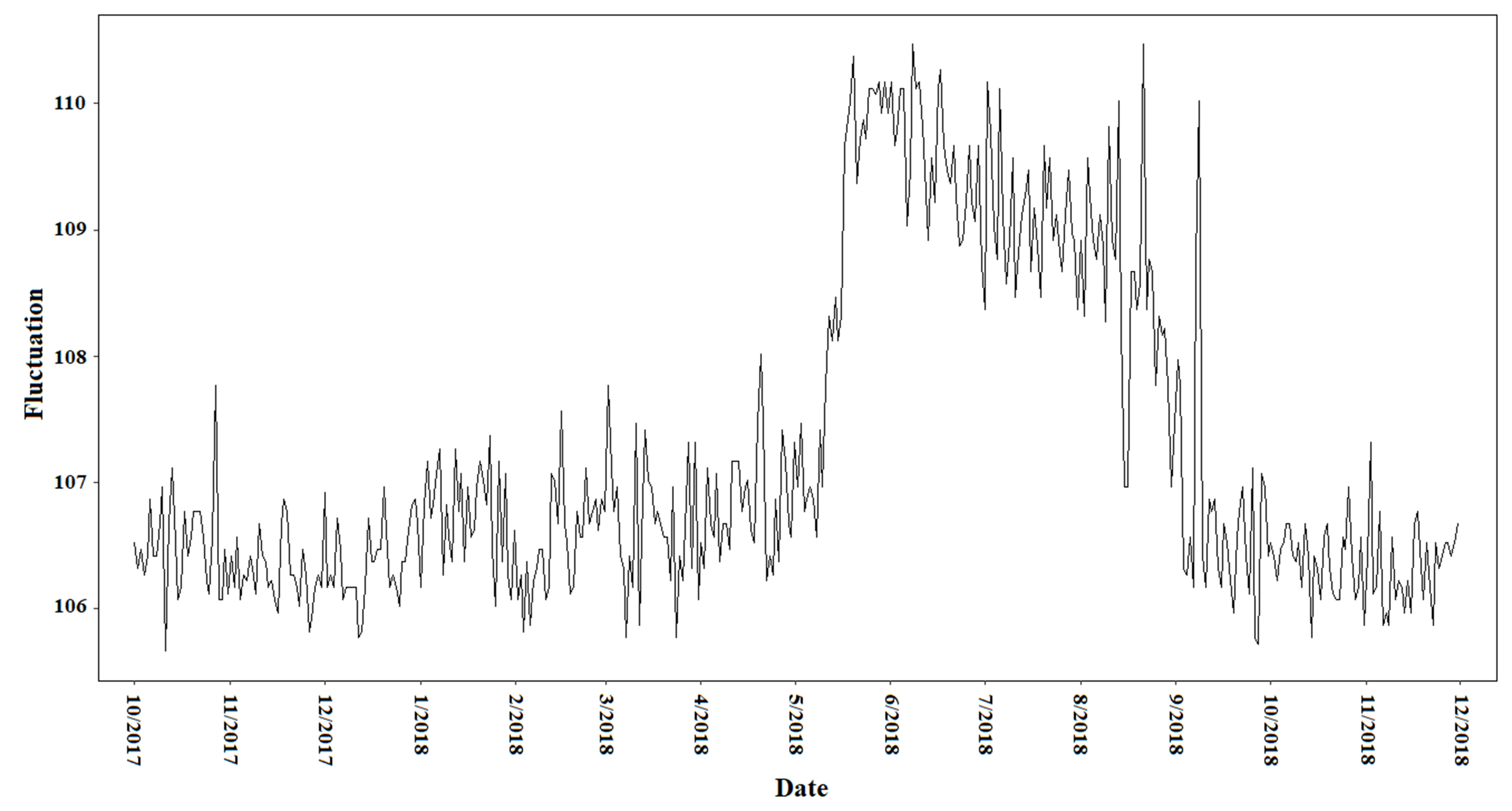

Supplement: Supplementary file 1 [file plants-10-02712-s001.zip › Figure S4.tif]
